# Variation in use of Caesarean section in Norway: An application of spatio-temporal Gaussian random fields

**DOI:** 10.1177/14034948211008579

**Published:** 2021-05-03

**Authors:** Janne Mannseth, Geir D. Berentsen, Hans J. Skaug, Rolv T. Lie, Dag Moster

**Affiliations:** 1 Department of Global Public Health and Primary Care, University of Bergen, Norway; 2 Department of Business and Management Science, Norwegian School of Economics, Norway; 3 Department of Mathematics, University of Bergen, Norway

**Keywords:** Spatial correlation, geographical variation, Caesarean section, SPDE, INLA, TMB

## Abstract

**Aims:**

Caesarean section (CS) is a medical intervention performed in Norway when a surgical delivery is considered more beneficial than a vaginal. Because deliveries with higher risk are centralized to larger hospitals, use of CS varies considerably between hospitals. We describe how the use of CS varies geographically by municipality. Since indications for CS should have little variation across the relatively homogenous population of Norway, we expect fair use of CS to be evenly distributed across the municipalities.

**Methods:**

Data from the Medical Birth Registry of Norway were used in our analyses (810,914 total deliveries, 133,746 CSs, 440 municipalities). We propose a spatial correlation model that takes the location into account to describe the variation in use of CS across the municipalities. The R packages R-INLA and TMB are used to estimate the yearly municipal CS rate and the spatial correlation between the municipalities. We also apply stratified models for different categories of delivering women (Robson groups). Estimated rates are displayed in maps and model parameters are shown in tables.

**Results:**

The CS rate varies substantially between the different municipalities. As expected, there was strong correlation between neighbouring municipalities. Similar results were found for different Robson groups.

**Conclusions:**

The substantial difference in CS use across municipalities in Norway is not likely to be due to specific medical reasons, but rather to hospitals’ different policies towards the use of CS. The policy to be either more or less restrictive to CS was not specific to any category of deliveries.

## Introduction

Caesarean section (CS) is in worldwide use as an important and sometimes life-saving medical procedure. Globally, the CS rates have risen markedly during the last decades [1]. The practice of CS varies considerably between countries and also between regions within countries [2,3]. The World Health Organization (WHO) has stated that at a population level, CS rates higher than 10% are not associated with improvements in maternal or infant mortality [4]. They also stated that CS is a procedure with possible risk of complications for both mother and child that should be performed only when medically necessary. Many countries have CS rates far beyond the recommended level.

In Norway, the national CS rate is now about 16% [5] with substantial differences in CS rates between apparently similar hospitals. Women typically give birth at a hospital close to their residence. A medical reason needs to be given for performing a CS, and the decision in each case is made by the practitioners at the hospital. Women with high-risk deliveries may be transferred to one of the larger hospitals if there is time to do so. Geographical variation in the proportion of high-risk deliveries may cause geographical variation in CS rates. Differences in obstetric preference, the geographical structure of the hospital system, and the distance from a woman’s residence to the hospital may, however, be more likely explanations. Such differences may be a challenge to Norway’s free, fair and universal healthcare system.

In this paper, we aim to provide a thorough geographical overview of CS use in Norway. We use a spatio-temporal model that includes random effects to describe the variation across municipalities of residence for the population. The model enables estimation of how the spatial correlation between selected observation locations is behaving as well as the CS rate for all observation locations in any given year of the study period. We present this method as a statistical tool for studying spatial variation, applicable to many situations in addition to that addressed in this paper.

If there are differences in CS use across the country, it is not unlikely that the differences are more pronounced for certain categories of deliveries. Robson groups are commonly used to monitor CS rates in categories based on common criteria for a CS delivery. We describe variation in CS use for the two common categories ‘spontaneous onset, term, singleton, cephalic, first-births’ (Robson group 1) and ‘spontaneous onset, term, singleton, cephalic, higher order-births without previous CS’ (Robson group 3). In addition, we describe the variation in CS use for two particularly interesting categories: deliveries of women with a previous CS (Robson group 5) and first births with a breech presentation (Robson group 6). Robson groups, also those not included in this study, are defined in Supplementary Table S1.

A description of the original data set, the data cleaning process and the development of the statistical model for CS use are presented in the Methods section. The Results section shows estimated covariate effects, model parameters and maps of the estimated municipality CS rates. In addition, estimates of the distribution of CS in four Robson groups are provided. Maps for the Robson group CS rates, details about the model, as well as the theory of the spatial parts such as the spatial effects are explained in the Supplementary material.

## Methods

### CS data

The data are obtained from the Medical Birth Registry of Norway (MBRN), which stores information on pregnancy and births in Norway and is based on compulsory notification. Starting in the year 1967, the registry contains data on every birth, stillbirth and late abortion after gestational age (GA) of 15 weeks [6]. In addition, relevant information such as parents, complications during delivery and mother’s health during pregnancy are recorded.

The MBRN data used in this paper includes information on labour type (vaginal delivery or CS), birth year, mother’s municipality and various outcomes associated with birth and CS (for mother and/or child). We also use information necessary to classify delivering women into the Robson groups 1, 3, 5 and 6, described in the Introduction. These groups combined capture 51.2% of the CSs in the data. Supplementary Table S1 reports how the CSs are spread between the Robson groups.

The study included 840,627 births from 1 January 2001 to 31 December 2014. In total, there were 138,394 CS deliveries. Births occurring in municipalities with 49 births or less per year were excluded (17 municipalities with a total of 1365 births), since many very low numbers would complicate the statistical analysis. We also excluded 3881 home deliveries, since those were not eligible for CS. Furthermore, births given by young (less than 15 years) or old (greater than 45 years) mothers were excluded (*n* = 2868 births), since they were very atypical, and we excluded 3859 abortions and 3951 births before 24 weeks of gestation since they were not eligible for CS. On the Svalbard group of islands, 222 births were also excluded because they were outside the geographical area of interest. In addition, births with missing information on mother’s municipality (*n* = 730), mother’s age (*n* = 104), GA (*n* = 4454), sex (*n* = 5656), birth weight (*n* = 8683), birth unit (*n* = 3589) or presentation (*n* = 3635) were also removed since they lacked critical information. Some observations had more than one type of missing value. Finally, we were left with a total of 810,914 births (133,746 CSs) from 440 municipalities of residence within the given time period as a basis for the study. A flowchart describing this process is found in the Supplemental Figure S7.

For each municipality we obtained the coordinates of the center of the municipality. This location was used to represent the location of all residents in that municipality. The length of Norway is 1752 km and its width 420 km, which is underlying the interpretation of some results regarding spatial correlation.

### Statistical methods

Some of the 440 municipalities in Norway used in our analysis have a low number of inhabitants and a low annual number of births (after removing those with < 50 births per year). Municipalities with few births can experience extreme CS proportions in a particular year. However, most municipalities have a higher number of inhabitants with a relatively consistent number of births each year. In the estimation process, the Gaussian random field approach that is explained in the Supplementary material in effect performs a smoothing by taking into account the use of CS in neighbouring municipalities. The estimated CS rates for the small municipalities will, therefore, be less affected by extreme values.

**Figure 1.**
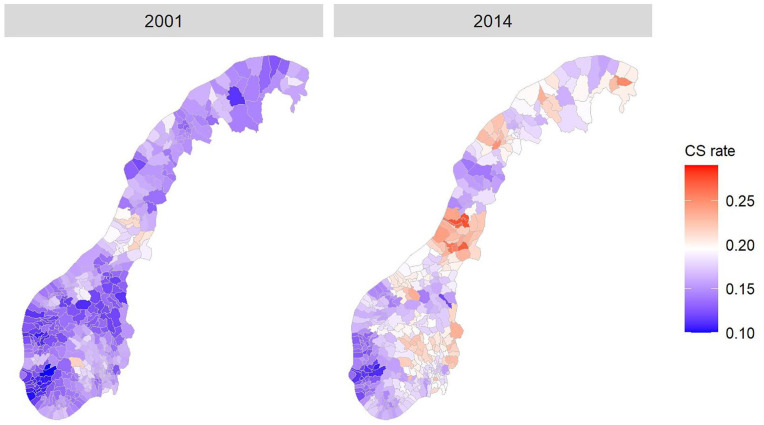
Estimated Caesarean section rates in Norway by municipality for years 2001 and 2014 (using reference value for all covariates).

A logistic regression model was used to relate individual birth outcomes (CS or not) to covariates and spatial and temporal trends. Covariates like maternal smoking, parity and breech presentation were treated as fixed effects. The covariates that were included were assumed to be associated with CS [7]. Random effects were used to describe spatial variations. The spatial (geographical) random effect was represented by a smooth surface which at each location (municipality) on a logit scale had a normal distribution with mean zero and variance σ_ϵ_2 . Hence, the spatial trend is a deviation from the overall mean, which for a given birth is determined by individual-specific covariates. Examples of the resulting geographically varying CS probability are shown in Figure 1. The smooth nature of the underlying surface is partly hidden by the fact that the figure assigns a constant value (that of the municipality centre) to the whole municipality.

The spatial surface is taken to be isotropic, meaning that the correlation between two municipalities was determined only by geographical distance, and not by their absolute locations. This assumption allows us to estimate a correlation range, *ρ* [8]. This is the distance in kilometres one must move away from a location before the spatial correlation has dropped below 0.1. This means that the spatial correlation at this distance is very small and no longer relevant. Spatial surfaces with a large *ρ* will look smooth, while small *ρ* give rise to wiggly surfaces. In the limit that *ρ* goes to zero, the spatial random effect collapses to a municipality specific random effect (which is not explicitly included in the model). The magnitude of the spatial effect is controlled by the standard deviation σ_ϵ_, which acts on the logit scale, as stated earlier.

The temporal trend, also acting on the logit scale, has two components. First, there is a global fixed-effect linear time trend, with an annual increase of *ω*, which equally affects all municipalities. In addition, for each municipality there is a random effect time trend, taken to be a zero-mean autoregressive process with correlation parameter *α*. Hence, for a given municipality, the correlation (logit scale) between two consecutive years is *α*. As for the spatial trend, the temporal trend only represents a deviation from the overall mean. The temporal and spatial trends are not modelled entirely separately, and should be thought of as a joint spatio-temporal random effect. Their interaction is such that the total marginal variance resulting from both spatial and temporal variation is σ_ϵ_2 /(1-α2). We assume temporal stationarity so that this variance does not change with time. Further, the interpretation of *ρ* as a spatial correlation distance (within each year) is preserved also when the temporal variation is included.

Fixed effects (*β*) associated with covariates together with the model parameters *ω*, σ_ϵ_, *ρ* and *α* were estimated by maximum likelihood using the R package TMB [9]. TMB obtains standard deviations of parameters using standard statistical theory, while it uses empirical Bayes estimates of the spatio-temporal trends. The R package R-INLA was used to set up an approximate spatial correlation matrix, using the stochastic partial differential equation (SPDE) approach [8,10,11]. More details about the computations are given in the Supplemental material.

The odds ratio (OR) associated with a fixed effect *β* is defined as exp(*β*). If an OR = 1, then *β* = 0, and the presence of the given covariate does not seem to affect the risk of CS. If the OR< 1 or the OR > 1 the presence of the given covariate is associated with a lower or higher risk of CS, respectively.

## Results

### Model parameters

**Figure 2.**
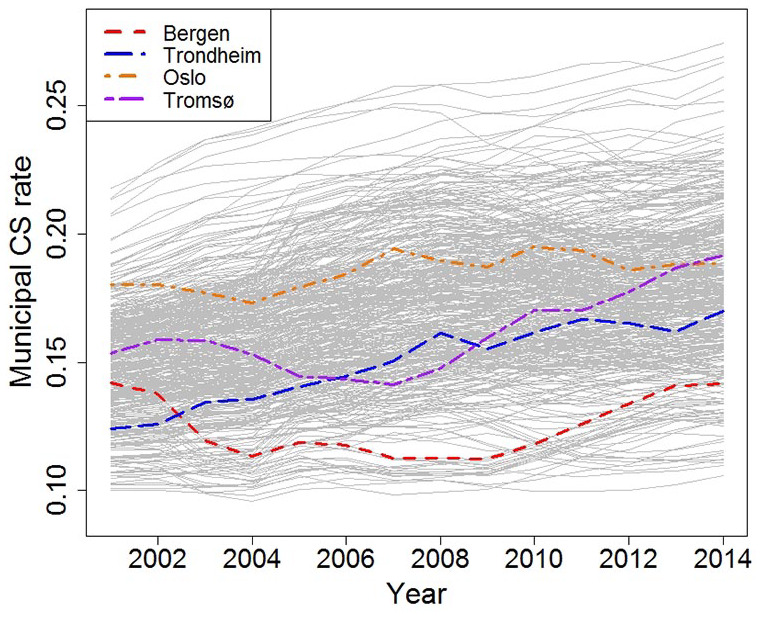
Estimated Caesarean section rates by year for the 440 Norwegian municipalities (grey), with four large cities highlighted.

Table I shows the estimated values of the spatial and temporal parameters for the main analysis. The estimated global (all municipalities) linear time effect, *ω*, is 0.024, consistent with the increasing time trend that is seen for CS in Norway [4]. When the global time trend has been removed, the estimated intra-municipal temporal correlation is estimated at *α* = 0.92. This high value indicates a strong correlation in time. Figure 2 shows the development in CS rate throughout 14 years for each of the 440 municipalities. The largest cities are highlighted and show a clear difference in CS rate. The standard error of the spatial random effect (σ_ϵ_) is estimated to 0.13 on a logit scale, which indicates a considerable variation in CS rates across the municipalities. On the real scale this amounts to a standard error of 0.025 for a CS probability of 0.25. The latter standard error controls how much geographical variation is allowed in Figure 1.

**Table I.** Estimated parameters with standard errors (SD) for spatial and temporal parameters.

Parameter	Value	SD
Spatial standard deviation (*σ*_ε_)	0.125	0.008
Correlation distance (*ρ* in km)	114.0	15.3
Temporal correlation (*α*)	0.920	0.020
Linear time trend (*ω*)	0.024	0.005

The value of the correlation distance parameter, *ρ*, is estimated at 114 km. As already said, this means that including all covariates, the distance (radius) at which the spatial correlation is in practice not relevant is around 114 km. In Norway, moving 114 km in any direction usually covers several municipalities. Therefore, this result indicates the presence of spatial correlation in CS between neighbouring municipalities.

We study the effect the covariates have on the risk of delivery by CS. These covariates are included to adjust for potential confounding from their geographical variation. Table II shows the estimated ORs with standard errors for the covariates. Breech presentation and having had CS previously are the two covariates with the highest ORs of 14.40 and 14027 respectively. Pregnancy complications (diabetes, hypertension, pre-eclampsia or eclampsia, placenta praevia or placental abruption) had the third highest OR of 3.36.

**Table II.** Estimated fixed effects of factors associated with Caesariasn section (CS) with standard errors. The odds ratio (OR) is the exponential of an effect of the logistic scale.

Associated factor	OR	SD
Maternal smoking in the beginning of pregnancy (ref: no smoking in the beginning of pregnancy)	1.12	0.018
Maternal smoking at the end of pregnancy (ref: no smoking at the end of pregnancy)	1.00	0.020
Maternal age (years)		
15–19	0.57	0.016
20–34	Ref	
35–44	1.70	0.015
Parity		
Nulliparous	2.71	0.022
Multiparous without previous CS	Ref	
Multiparous with previous CS	14.27	0.145
Gestational age (weeks + days)		
< 37 + 0	2.52	0.030
37 + 0 – 41 + 6	Ref	
⩾ 42 + 0	1.59	0.022
Volume maternity unit		
Small	0.96	0.017
Medium	Ref	
Large	0.96	0.013
Induction of birth (ref: no induction)	0.88	0.008
Complication during pregnancy/delivery (ref: no complication during pregnancy/delivery)	3.36	0.039
Sex (ref: male)	0.88	0.006
Macrosomia: > 4500 g (ref: ⩽ 4500 g)	2.09	0.035
Breech presentation (ref: no breech presentation)	14.40	0.192

### Geographical pattern

Together with the parameter estimates, the fitted geographical pattern of CS rates in the municipalities is the main result of the analysis. Figure 1 shows the estimated municipality CS rates for the first and last year of the study, when the national CS rates were 15 and 17.2% respectively (note for later that the colour scales differ from figure to figure). In 2001, all estimated rates are well below 0.2. In 2014, the maximum CS rate is estimated to exceed 0.25. The areas that in 2001 are estimated to have the highest CS rates, are the same areas that have the highest CS rates in 2014. This clearly corresponds to the general national (and international) increase for CS. Furthermore, the colours in the map of both the year 2001 and 2014 in Figure 1 show strong clustering, meaning that red municipalities are surrounded by other red municipalities (for instance in the east and middle of the map) and purple municipalities of other purple ones (for instance in the west of the map). This indicates that the CS rates in neighbouring municipalities are correlated, and one might suspect that this is partly because they tend to be served by the same hospitals.

The estimated CS rates in Figure 1 are not compared visually with the CS rates that can be extracted directly from the data. Because of the issue with extreme values that was discussed in the Methods section, a graphical comparison can be difficult. Our model can be considered as a smoothing of the empirical data in both time and space.

### Robson groups of CS

Several clinical criteria such as GA and parity (Table II) have an effect on the CS probability and are used to divide pregnancies into Robson groups. The overall spatial variation of CS may, to a large degree, to be due to differences in clinical practice. By fitting separate models for each Robson group it should be possible to see whether a geographical difference in clinical practice is general or restricted to practice for particular Robson groups. The four groups we have used are defined in the Introduction as well as more thoroughly in the Supplemental material (Table S1). The particular Robson groups included in the analysis are chosen based on prevalence (groups 1, 3 and 5) and interest in studying a group with a high CS rate (group 6).

The model used for performing the stratified analyses for the Robson groups is the same as the one used for the main analysis, described in the Methods section. However, some of the covariates from Table II were excluded from the stratified analyses, due to the fact that they are used to define the Robson groups (parity, GA, induction of birth and breech presentation).

Table III shows the estimated parameter values for the Robson groups. These can be compared with the results for the overall CS model in Table I. The linear time trend *ω* is slightly positive for all the Robson groups, which is in correspondence with the general increase in CS use. The parameter *α*, the autocorrelation, is around 0.9 for all the groups and shows a strong correlation in time. The standard deviation of the spatial random effect, σ_ϵ_, shows substantial variation in the CS rates between municipalities. For the spontaneous onset, term, singleton, cephalic, first-births (Robson group1) and spontaneous onset, term, singleton, cephalic, higher order-births without previous CS (Robson group 3), the variation was a little higher than for the common CS model, 0.16 and 0.17 respectively. Women with a previous CS (Robson group 5) had the smallest variation with 0.09, while first births with a breech presentation (Robson group 6) interestingly had the largest variation with 0.32. The parameter *ρ* was large for all the Robson groups and covers multiple municipalities with its radius. This means that there is a strong spatial correlation within all of the four Robson groups.

**Table III.** Estimated parameter values with standard errors for spatial and temporal parameters for each Robson group.

Parameter	Robson 1		Robson 3		Robson 5		Robson 6	
	Value	SD	Value	SD	Value	SD	Value	SD
Spatial standard deviation (*σ*_ϵ_)	0.159	0.016	0.170	0.024	0.085	0.015	0.317	0.043
Correlation distance (*ρ* in km)	124.7	22.0	107.1	23.0	146.4	44.7	139.5	33.5
Temporal correlation (*α*)	0.852	0.031	0.913	0.025	0.940	0.022	0.887	0.035
Linear time trend (*ω*)	0.036	0.007	0.051	0.009	0.028	0.006	0.024	0.018

Figures S1–S4 are included in the Supplemental material. They show the estimated municipal CS rates for each of the four Robson groups for the year 2001 and the year 2014, and are described in this paragraph. As already mentioned, the colour scales differ from figure to figure. For Robson group 1 (Figure S1), both year 2001 and year 2014 show the same main geographical structure as seen in Figure 1, where the west and south of Norway had the lowest CS rates and the eastern and middle/northern parts had the highest CS rates. There was also a very clear increase in CS from the first to the last year of the study. For Robson group 3 (Figure S2) we found that the highest CS rates were very small, around 0.05. However, a similar pattern as in Figure 1 was still present for both years, having the highest rates in the eastern and middle/northern parts of the country. Robson group 5 (Figure S3) had quite high CS rates in most regions. Still, the difference between the highest and smallest CS rates for this group were around 20% and the same regions as in Figure 1 stood out. Robson group 6 (Figure S4) included less than 2% of all births in the data (Table S1). There are consistently quite high CS rates for this group in Norway, but nevertheless the same geographical pattern is present for this group as well. In addition, the western part of the country stands out with very low rates compared to the rest of the country and also with a decrease rather than the general increase from the year 2001 to the year 2014.

## Discussion

We have fitted a model with CS rate in mother’s municipality of residence as the response and estimated effects of covariates, temporal and spatial parameters. The estimated covariate effects are in line with what we expected and are not our main focus. Estimates of the remaining or ‘unexplained’ geographical variation in the use of CS is shown in a map of the municipalities. Analyses were done both for the total CS rate, and for the rates of CS in four Robson groups with spontaneous onset of delivery. In the total CS analysis, we estimated an overall increase in CS rate from the first to the last year of the study period. Within each time-period, the estimated CS rates varied systematically and considerably between different parts of Norway. Women in many municipalities were estimated to have CS rates much higher than those recommended by WHO.

The estimates of the variance parameter σ_ϵ_ confirmed that there was considerable variation between municipalities, and the correlation distance *ρ* showed that neighbouring municipalities in a region, as expected, tended to have similar rates. Together, these factors show that there was a systematic pattern of spatial correlation for the CS procedure. One may argue that the spatial correlation should not be a function of geographical (Euclidean) distance only, and that ‘driving distance’ is a more relevant metric. Municipalities in the east and middle north had the highest CS rates, while municipalities in the western areas had the lowest CS rates. It is also interesting that an area of municipalities in the northern part of the country had lower CS rates compared to municipalities just south and north of them. This is likely to reflect a difference in the practice of local hospitals, rather than a difference in local risk factors associated for use of CS. Further, there was a strong inter-annual correlation within each municipality in addition to the overall time trend. This implies that deviation from the overall time trend is persistent within each municipality.

We stratified the analyses using four key Robson groups. The estimates of the temporal and spatial parameters were similar to those of the total CS model, with correlation distances large enough to cover multiple municipalities for all four groups. Because of their different sizes, the range of the average CS rates was different between the Robson groups. However, we still found that the largest and smallest CS rates within each Robson group belonged to the same areas as for the total CS analysis. We did not find any evidence that the geographical variation of CS for Robson groups had a different pattern than for the overall CS pattern. Areas with lower total CS rates also had lower CS rates for the Robson groups. Fitting the spatio-temporal model for the smallest Robson groups would be difficult and lead to estimates with high uncertainty. The models are best suited for outcomes that are not too rare.

Our study had the strength of data with a consistent method of collection and complete population coverage due to compulsory reporting. In Norway, public healthcare and pregnancy and labour services are free of charge. Regarding CS, this means that the financial factor is irrelevant for a delivering woman. We were able to track mother’s municipality of residence with high precision. Our analyses made it possible to show that there is huge unexplained variation in the likelihood that a regular pregnant woman in a specific municipality of residence is delivered by CS and that this variation is likely to be caused by differences in hospital’s obstetric preferences for CS.

Our analysis was limited by the crudeness of the geographical information on the residence. We used a common location for a whole municipality, and addresses and coordinates of each residence would have been an improvement. Such information was, unfortunately, not available in the registry data. We excluded a small number of births that were either not eligible for CS, lacked critical information or were outside mainland Norway. This should not have affected our results.

Other studies have established variation in CS rates across geographical units. In a recent paper it was found that CS rates vary between hospitals in Florida [10]. The paper explains some of the variation with the cultural practice for mothers belonging to certain subgroups combined with hospital practice. Another study considered geographic and temporal variation in CS rates in China [11]. Here, considerable geographic variation between different counties was found. The national CS rate ranges from 28.8 to 34.9% in the study period. The rates were typically the lowest and the highest in rural and urban areas respectively, ranging from less than 10 to over 60%. A recent study from Denmark found clear between hospital variation in CS rates [7]. The variation was unexpected, and the study suggests that it could be connected to systematic differences in hospital practice. These studies all find geographic variation in CS rates, but not on the level of residence. The Danish study was more relevant for our study because of important similarities in the healthcare system and socioeconomic factors in general. It also addressed the phenomenon that referrals from smaller to larger hospitals affect the CS rates. Our study avoids the complications of comparing hospitals by using mother’s municipality of residence in the geographical analysis. The hospital infrastructure is complex and there may be good reasons why hospitals are different. Women in Norway and Denmark should, however, be offered a similar standard of obstetric care independent of their area of residence.

In conclusion, our analysis suggests that differences between hospitals’ CS practice create large variations in CS rates between different areas of residence. Similar geographical variation was seen for Robson groups, which suggest that the unequal use of CS is driven by a more or less liberal policy towards the CS procedure itself and has no specific medical reason. Our description of the variation in use of CS did not attempt to determine whether it is more beneficial for a woman to live in a municipality with a high or low CS rate. This may be an important question for future research.

## Supplemental Material

sj-jpeg-1-sjp-10.1177_14034948211008579 – Supplemental material for Variation in use of Caesarean section in Norway: An application of spatio-temporal Gaussian random fields Click here for additional data file. Supplemental material, sj-jpeg-1-sjp-10.1177_14034948211008579 for Variation in use of Caesarean section in Norway: An application of spatio-temporal Gaussian random fields by Janne Mannseth, Geir D. Berentsen, Hans J. Skaug, Rolv T. Lie and Dag Moster in Scandinavian Journal of Public Health

sj-jpeg-2-sjp-10.1177_14034948211008579 – Supplemental material for Variation in use of Caesarean section in Norway: An application of spatio-temporal Gaussian random fields Click here for additional data file. Supplemental material, sj-jpeg-2-sjp-10.1177_14034948211008579 for Variation in use of Caesarean section in Norway: An application of spatio-temporal Gaussian random fields by Janne Mannseth, Geir D. Berentsen, Hans J. Skaug, Rolv T. Lie and Dag Moster in Scandinavian Journal of Public Health

sj-jpeg-3-sjp-10.1177_14034948211008579 – Supplemental material for Variation in use of Caesarean section in Norway: An application of spatio-temporal Gaussian random fields Click here for additional data file. Supplemental material, sj-jpeg-3-sjp-10.1177_14034948211008579 for Variation in use of Caesarean section in Norway: An application of spatio-temporal Gaussian random fields by Janne Mannseth, Geir D. Berentsen, Hans J. Skaug, Rolv T. Lie and Dag Moster in Scandinavian Journal of Public Health

sj-jpeg-4-sjp-10.1177_14034948211008579 – Supplemental material for Variation in use of Caesarean section in Norway: An application of spatio-temporal Gaussian random fields Click here for additional data file. Supplemental material, sj-jpeg-4-sjp-10.1177_14034948211008579 for Variation in use of Caesarean section in Norway: An application of spatio-temporal Gaussian random fields by Janne Mannseth, Geir D. Berentsen, Hans J. Skaug, Rolv T. Lie and Dag Moster in Scandinavian Journal of Public Health

sj-jpeg-5-sjp-10.1177_14034948211008579 – Supplemental material for Variation in use of Caesarean section in Norway: An application of spatio-temporal Gaussian random fields Click here for additional data file. Supplemental material, sj-jpeg-5-sjp-10.1177_14034948211008579 for Variation in use of Caesarean section in Norway: An application of spatio-temporal Gaussian random fields by Janne Mannseth, Geir D. Berentsen, Hans J. Skaug, Rolv T. Lie and Dag Moster in Scandinavian Journal of Public Health

sj-jpeg-6-sjp-10.1177_14034948211008579 – Supplemental material for Variation in use of Caesarean section in Norway: An application of spatio-temporal Gaussian random fields Click here for additional data file. Supplemental material, sj-jpeg-6-sjp-10.1177_14034948211008579 for Variation in use of Caesarean section in Norway: An application of spatio-temporal Gaussian random fields by Janne Mannseth, Geir D. Berentsen, Hans J. Skaug, Rolv T. Lie and Dag Moster in Scandinavian Journal of Public Health

sj-jpeg-7-sjp-10.1177_14034948211008579 – Supplemental material for Variation in use of Caesarean section in Norway: An application of spatio-temporal Gaussian random fields Click here for additional data file. Supplemental material, sj-jpeg-7-sjp-10.1177_14034948211008579 for Variation in use of Caesarean section in Norway: An application of spatio-temporal Gaussian random fields by Janne Mannseth, Geir D. Berentsen, Hans J. Skaug, Rolv T. Lie and Dag Moster in Scandinavian Journal of Public Health

sj-tex-8-sjp-10.1177_14034948211008579 – Supplemental material for Variation in use of Caesarean section in Norway: An application of spatio-temporal Gaussian random fields Click here for additional data file. Supplemental material, sj-tex-8-sjp-10.1177_14034948211008579 for Variation in use of Caesarean section in Norway: An application of spatio-temporal Gaussian random fields by Janne Mannseth, Geir D. Berentsen, Hans J. Skaug, Rolv T. Lie and Dag Moster in Scandinavian Journal of Public Health

## References

[1] Betrán AP Ye J Moller A-B , et al. The increasing trend in Caesarean section rates: Global, regional and national estimates, 1990–2014. PloS one 2016;11:e0148343. 26849801 10.1371/journal.pone.0148343 PMC4743929

[2] Kozhimannil KB Arcaya MC Subramanian SV. Maternal clinical diagnoses and hospital variation in the risk of cesarean delivery: Analyses of a National US Hospital Discharge Database. PLoS medicine 2014;11:e1001745. 25333943 10.1371/journal.pmed.1001745 PMC4205118

[3] Bragg F Cromwell DA Edozien LC , et al. Variation in rates of Caesarean section among English NHS trusts after accounting for maternal and clinical risk: Cross sectional study. BMJ 2010;341:c5065. 20926490 10.1136/bmj.c5065 PMC2950923

[4] Betran AP Torloni MR Zhang JJ , et al. WHO Statement on Caesarean section rates. BJOG 2016;123:667–670. 26681211 10.1111/1471-0528.13526 PMC5034743

[5] Folkehelseinstituttet. Medical birth registry and abortion registry ‘Institusjonsstatistikk - Is4’. http://statistikkbank.fhi.no/mfr/ (accessed 20 August 2020).

[6] Irgens LM. The Medical Birth Registry of Norway: Epidemiological research and surveillance throughout 30 years. Acta Obstet Gynecol Scand 2000;79:435–439. 10857866

[7] Wehberg S Guldberg R Gradel KO , et al. Risk factors and between-hospital variation of Caesarean section in Denmark: A cohort study. BMJ open 2018;8:e019120. 10.1136/bmjopen-2017-019120 PMC5829888 29440158

[8] Lindgren F Rue H Lindström J. An explicit link between Gaussian fields and Gaussian Markov random fields: The stochastic partial differential equation approach. J R Stat Soc Series B Stat Methodol 2011;73:423–498

[9] Kasper K Anders N Casper WB , et al. TMB: Automatic differentiation and laplace approximation. J Stat Software 2016;70:1–21.

[10] Blangiardo M Cameletti M. Spatial and Spatio-temporal Bayesian Models with R-INLA. Chichester: John Wiley & Sons, 2015, pp. 194–196.

[11] Lindgren F Rue H. Bayesian spatial modelling with R-INLA. J Stat Software 1995;63. DOI: 10.18637/jss.v063.i19.

[12] Sebastião YV Womack L Vamos CA , et al. Hospital variation in Cesarean delivery rates: Contribution of individual and hospital factors in Florida. Am J Obstet Gynecol 2016;214:123-e1. 10.1016/j.ajog.2015.08.027 26292046

[13] Li H-T Luo S Trasande L , et al. Geographic variations and temporal trends in Cesarean delivery rates in China, 2008–2014. JAMA 2017;317:69–76. 28030701 10.1001/jama.2016.18663

