# Supplementary figures and images for: Variation in use of Caesarean section in Norway: An application of spatio-temporal Gaussian random fields
Source: Scand J Public Health. 2021 May 3;49(8):891–8. doi: 10.1177/14034948211008579 (PMC8573683; doi:10.1177/14034948211008579)

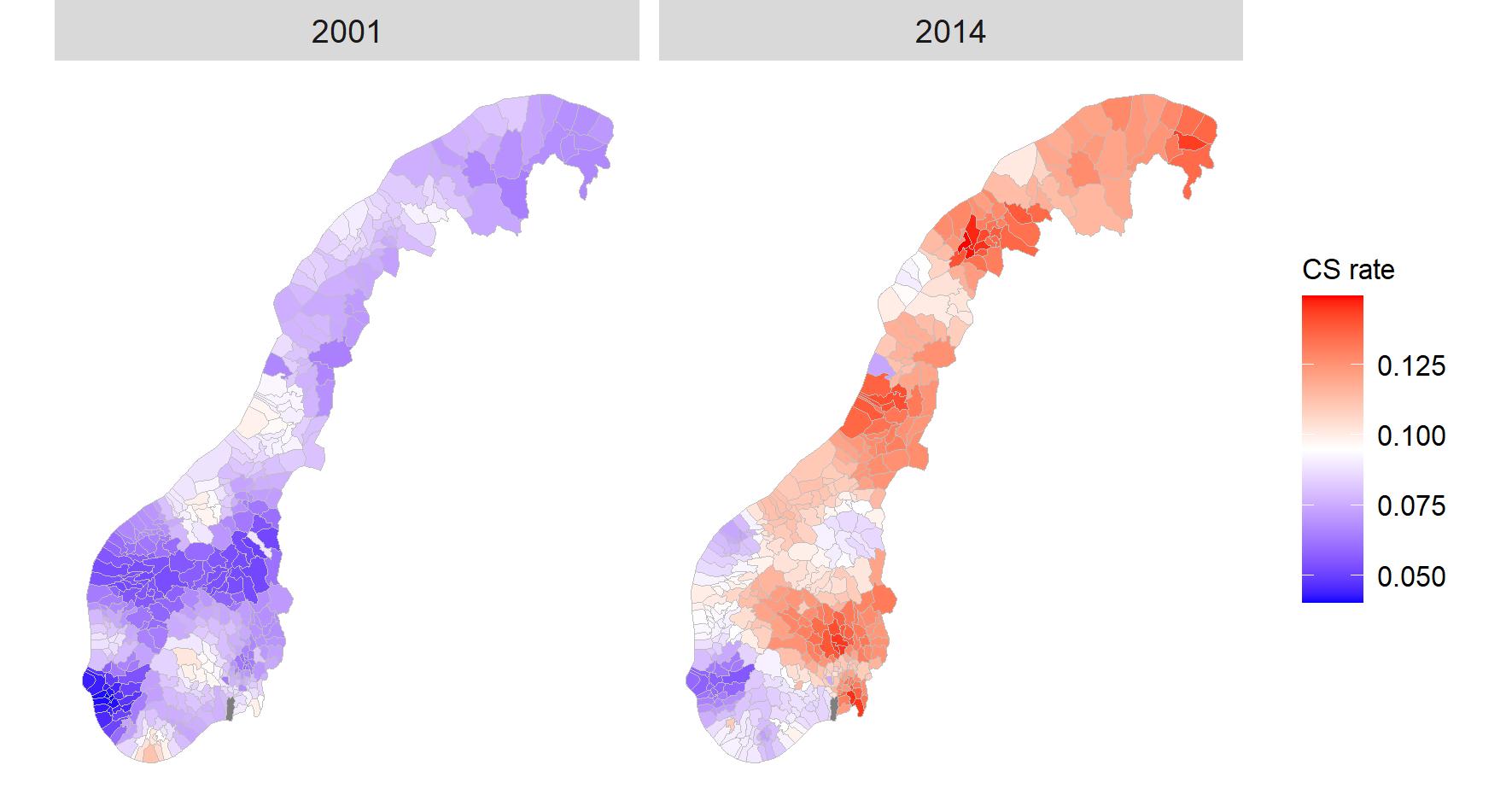

Supplement: sj-jpeg-1-sjp-10.1177_14034948211008579 – Supplemental material for Variation in use of Caesarean section in Norway: An application of spatio-temporal Gaussian random fields [file sj-jpeg-1-sjp-10.1177_14034948211008579.jpeg]

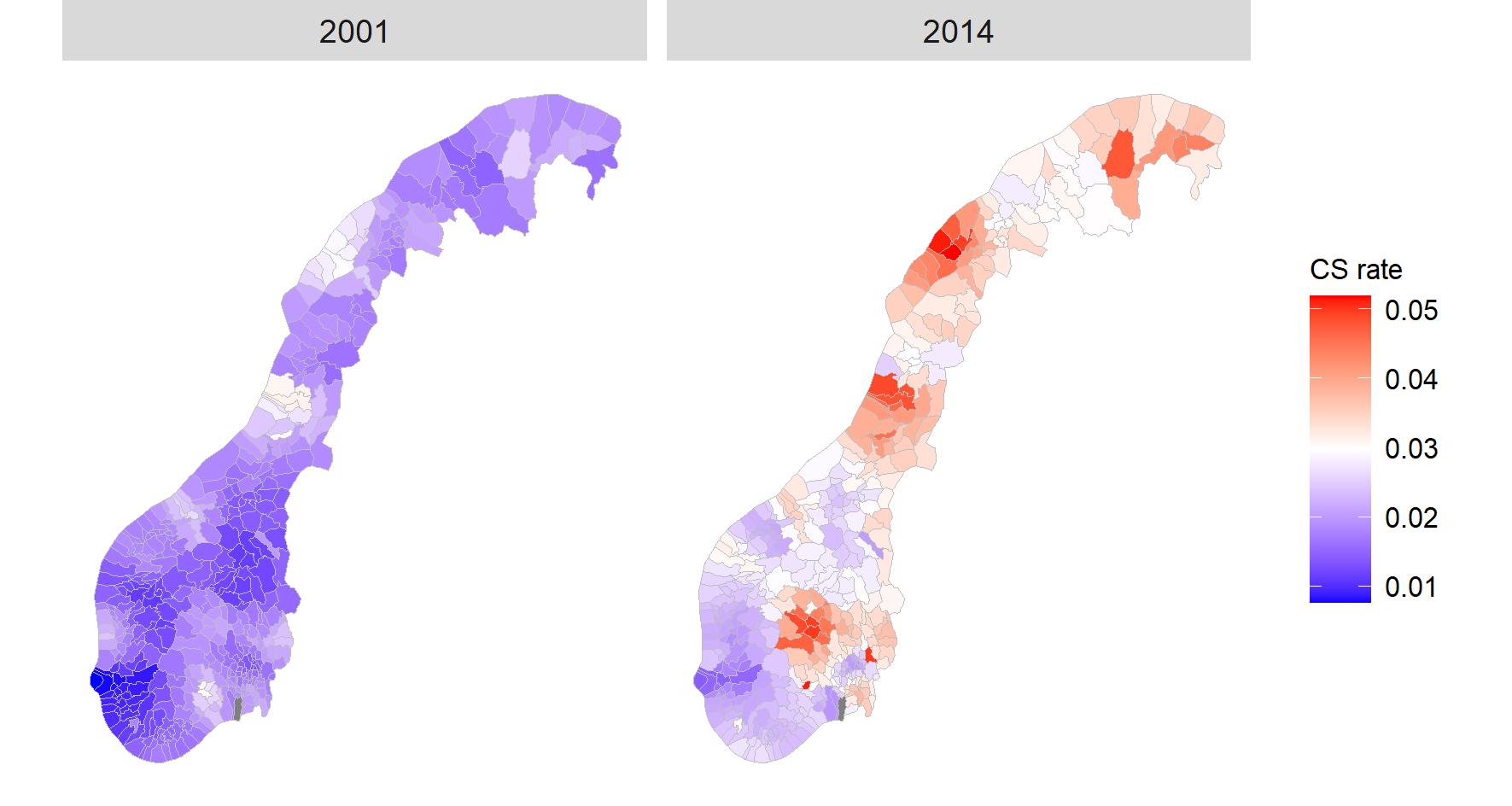

Supplement: sj-jpeg-2-sjp-10.1177_14034948211008579 – Supplemental material for Variation in use of Caesarean section in Norway: An application of spatio-temporal Gaussian random fields [file sj-jpeg-2-sjp-10.1177_14034948211008579.jpeg]

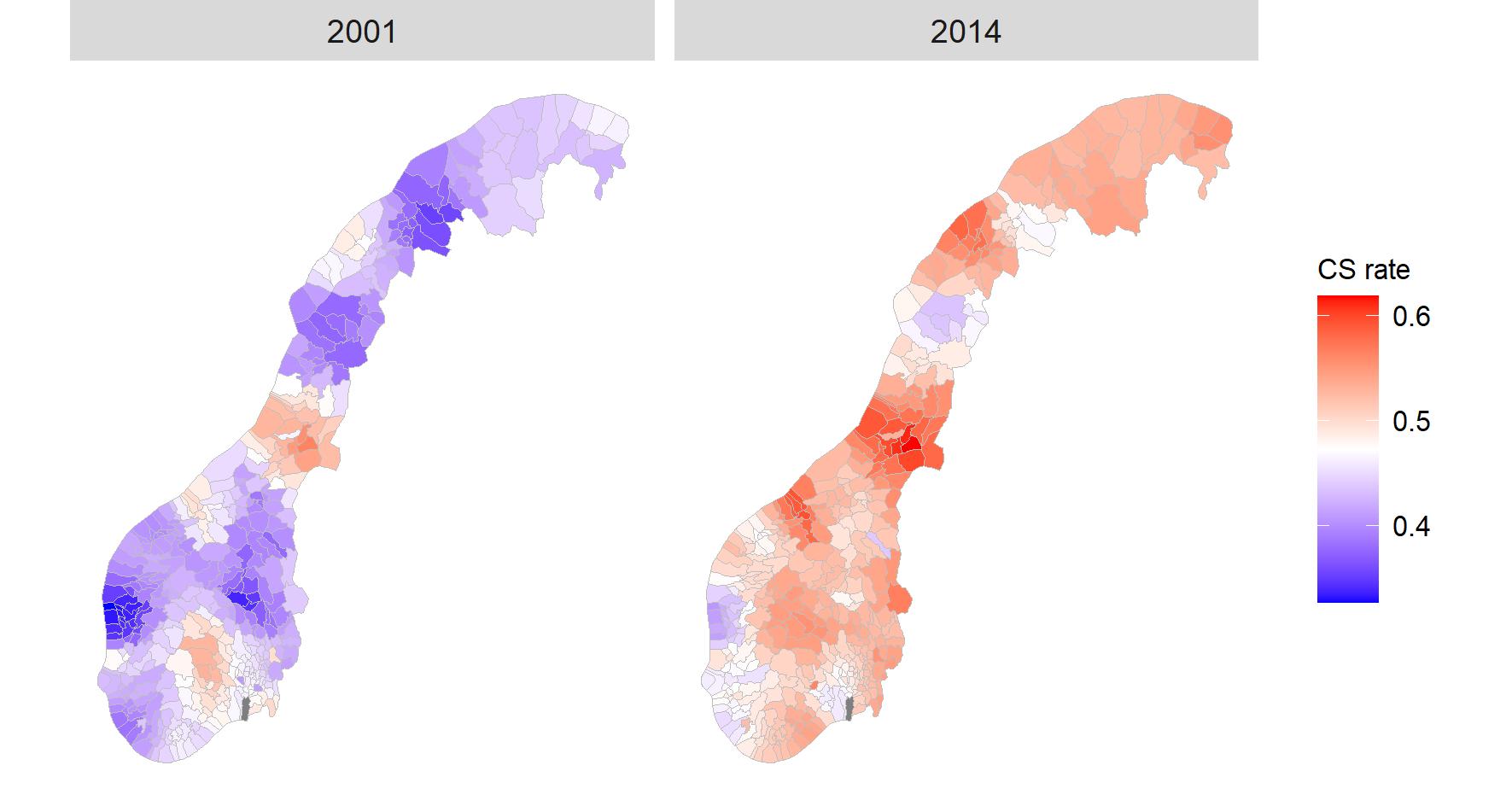

Supplement: sj-jpeg-3-sjp-10.1177_14034948211008579 – Supplemental material for Variation in use of Caesarean section in Norway: An application of spatio-temporal Gaussian random fields [file sj-jpeg-3-sjp-10.1177_14034948211008579.jpeg]

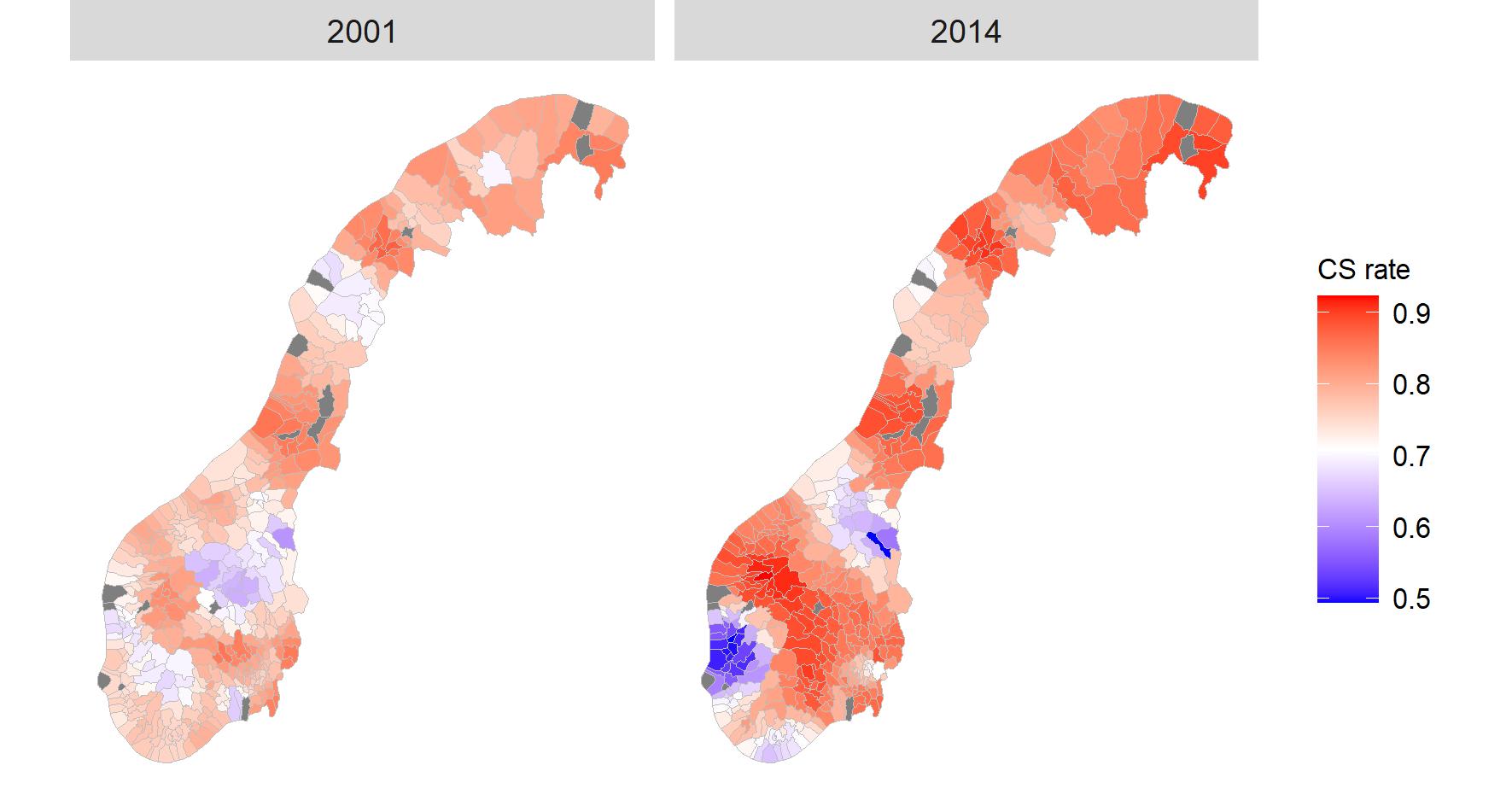

Supplement: sj-jpeg-4-sjp-10.1177_14034948211008579 – Supplemental material for Variation in use of Caesarean section in Norway: An application of spatio-temporal Gaussian random fields [file sj-jpeg-4-sjp-10.1177_14034948211008579.jpeg]

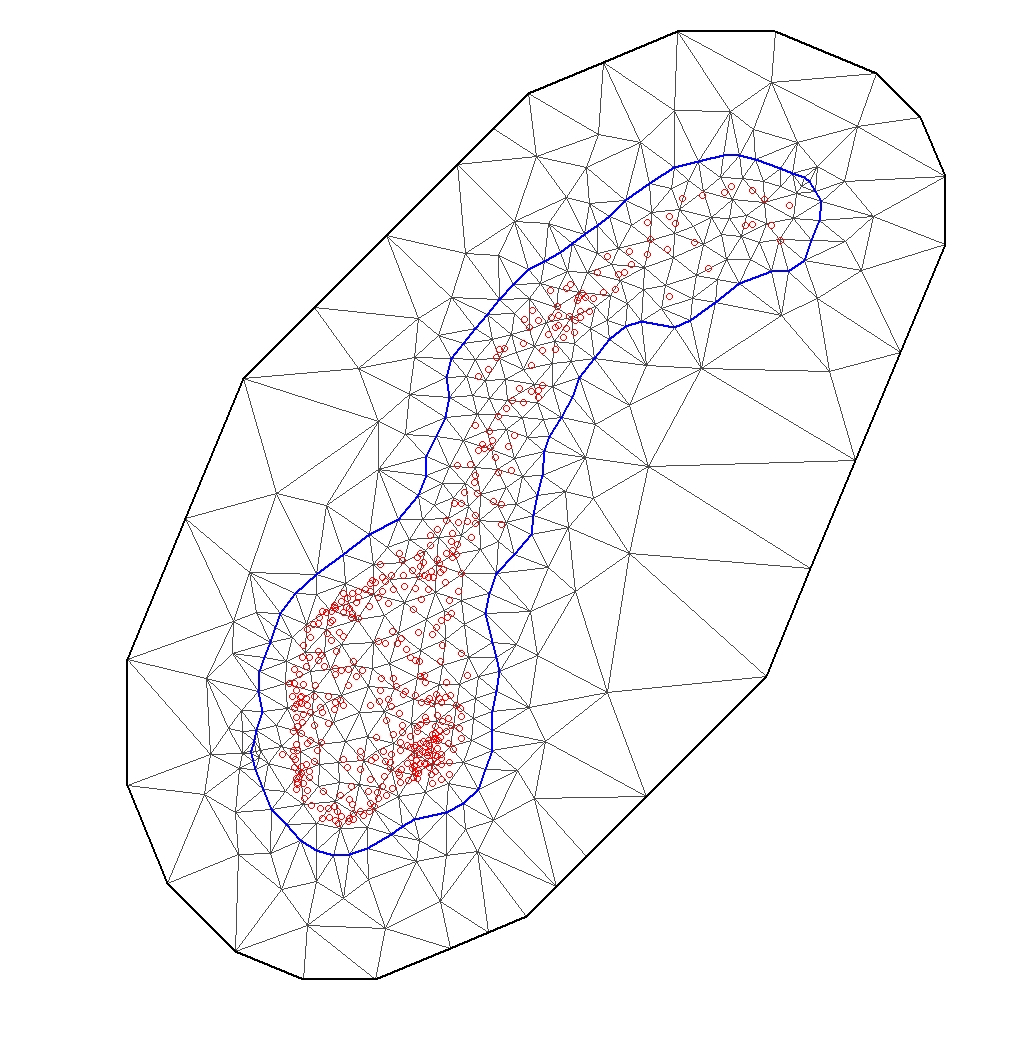

Supplement: sj-jpeg-5-sjp-10.1177_14034948211008579 – Supplemental material for Variation in use of Caesarean section in Norway: An application of spatio-temporal Gaussian random fields [file sj-jpeg-5-sjp-10.1177_14034948211008579.jpeg]

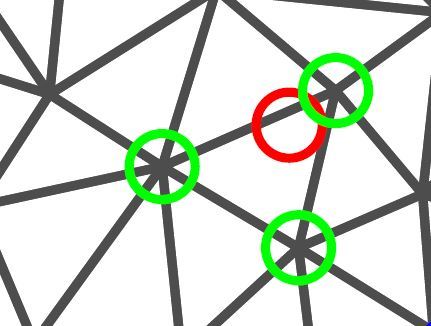

Supplement: sj-jpeg-6-sjp-10.1177_14034948211008579 – Supplemental material for Variation in use of Caesarean section in Norway: An application of spatio-temporal Gaussian random fields [file sj-jpeg-6-sjp-10.1177_14034948211008579.jpeg]

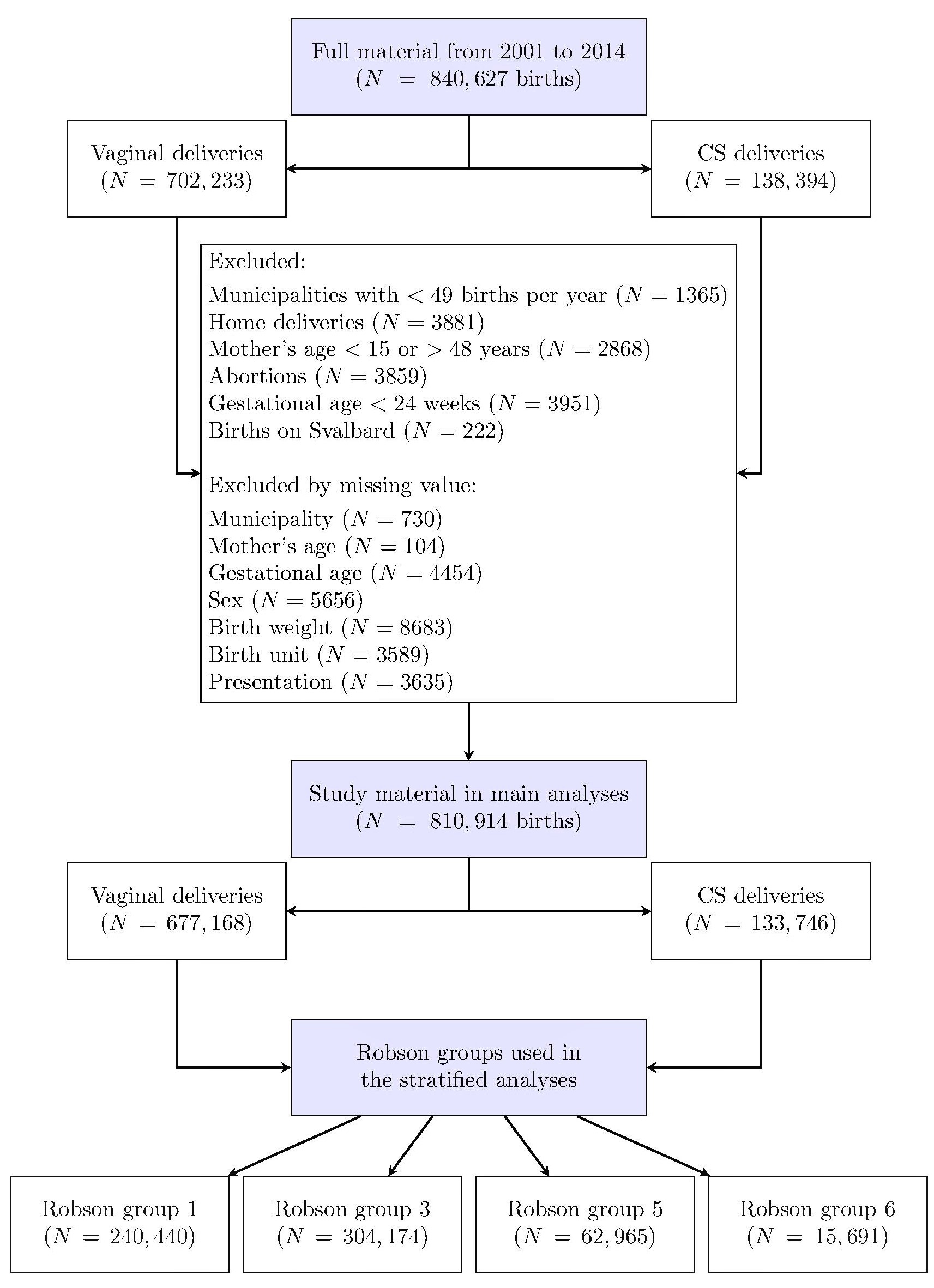

Supplement: sj-jpeg-7-sjp-10.1177_14034948211008579 – Supplemental material for Variation in use of Caesarean section in Norway: An application of spatio-temporal Gaussian random fields [file sj-jpeg-7-sjp-10.1177_14034948211008579.jpeg]
